# Border Habitat Effects on Captures of *Halyomorpha halys* (Hemiptera: Pentatomidae) in Pheromone Traps and Fruit Injury at Harvest in Apple and Peach Orchards in the Mid-Atlantic, USA

**DOI:** 10.3390/insects12050419

**Published:** 2021-05-08

**Authors:** James Christopher Bergh, William R. Morrison, Jon W. Stallrich, Brent D. Short, John P. Cullum, Tracy C. Leskey

**Affiliations:** 1 Virginia Tech, Alson Smith, Jr. Agricultural Research and Extension Center, Winchester, VA 22602, USA; 2 United States Department of Agriculture Agricultural Research Service, Center for Animal Health and Grain Research, Manhattan, KS 66502, USA; william.morrison@usda.gov; 3 Department of Statistics, Carolina State University, Raleigh, NC 27695, USA; jwstalli@ncsu.edu; 4 Trécé, Inc., Adair, OK 74330, USA; bshort@trece.com; 5 United States Department of Agriculture Agricultural Research Service, Appalachian Fruit Research Station, Kearneysville, WV 25430, USA; john.cullum@usda.gov (J.P.C.); tracy.leskey@usda.gov (T.C.L.)

**Keywords:** brown marmorated stink bug, monitoring, risk assessment, invasive species

## Abstract

**Simple Summary:**

Brown marmorated stink bug (BMSB) is a significant threat to the production of tree fruit, corn and soybean, and some vegetable crops in much of the USA and abroad. Its feeding causes injury that reduces crop quality and yield. BMSB invades crop fields from adjoining habitats, where it also feeds and develops on a broad range of wild and cultivated plants. Thus, it is considered a perimeter-driven threat, and research on management tactics to reduce insecticide applications against it has focused on intervention at crop edges. Woodlands often border one or more edges of crop fields in the Mid-Atlantic, USA, and are considered a main source of BMSB invasion, although tree fruit orchards in this region are typically also bordered along other edges by other habitats, including other tree fruit blocks and field crops. The effect of woodlands and other habitats bordering orchards on BMSB captures in pheromone traps and crop injury at harvest has not been compared. A two-year study in Mid-Atlantic fruit orchards confirmed that BMSB captures and fruit injury were often highest at edges bordering woodlands, but that other border habitats also contributed significantly to captures and injury in some instances.

**Abstract:**

The invasive *Halyomorpha halys* invades crop fields from various bordering habitats, and its feeding on crops has caused significant economic losses. Thus, *H. halys* is considered a perimeter-driven threat, and research on alternative management tactics against it has focused on intervention at crop edges. Woodlands adjacent to crop fields contain many hosts of *H. halys* and are therefore considered “riskiest” in terms of pest pressure and crop injury. However, tree fruit orchards in the Mid-Atlantic, USA, are often bordered on one or more sides by woodlands and other habitats, including other tree fruit blocks, and field crops. Monitoring *H. halys* using pheromone traps has most often focused on the crop–woodland interface, but the relative effects of woodlands and other habitats bordering orchards on pest pressure and crop injury have not been examined. A two-year study comparing seasonal captures of *H. halys* and fruit injury among different habitats bordering commercial apple and peach orchards in the Mid-Atlantic revealed that while woodland borders often posed the greatest risk, other border habitats also contributed significantly to captures and injury in numerous instances. The relevance of these findings to refining and optimizing perimeter-based monitoring and management approaches for *H. halys* is discussed.

## 1. Introduction

Feeding injury from the invasive brown marmorated stink bug, *Halyomorpha halys* (Stål) (Hemiptera: Pentatomidae), has impacted the production of numerous crops in the USA [1]. This has been especially pronounced in the Mid-Atlantic region, where a widespread and highly damaging outbreak in 2010 has been followed by ongoing issues associated with its management in tree fruits and other specialty crop systems, including the disruption of IPM practices from use of the broad-spectrum insecticides considered most effective against it [2,3]. Since 2010, *H. halys* has spread from the Mid-Atlantic to many other states and some Canadian provinces (stopbmsb.org), where it is causing similar issues or showing indications of the potential to do so. Moreover, *H. halys* has established in parts of Europe and the Caucasian region, where it is impacting tree fruit and tree nut production [4,5].

*Halyomorpha halys* adults and nymphs feed on a broad range of wild and cultivated plants [6,7,8] and are therefore widely distributed in rural and semi-urban landscapes. Both of these life stages exhibit considerable vagility and dispersal capacity [9,10,11,12], and both can cause economic crop injury [13]. Many tree fruit orchards in the eastern US are planted as essentially rectangular blocks that are often bordered on one or more sides by unmanaged woodlands that can serve as a reservoir for *H. halys* [1,7]. However, aside from woodland borders, most orchard blocks also have one or more of the following habitats along their other edges; other tree fruit blocks, row crops, field crops, fallow fields, or human-made structures within which adult *H. halys* can overwinter, often in large numbers. Moving between wild and cultivated hosts during the growing season, highest *H. halys* densities have been reported at the edges of crop fields next to woodlands and agronomic crops [14,15]. Moreover, feeding injury from *H. halys* to apples [16], soybeans, and corn [15] has often been highest at crop edges adjacent to woodlands. These wooded edges have therefore been considered “risky” borders because they can harbor large *H. halys* populations that can invade crops during the growing season.

For this reason, many *H. halys* monitoring studies using pheromone-baited traps have focused on the crop–woodland interface [17,18,19,20,21,22]. Moreover, given that *H. halys* is considered a perimeter-driven threat, investigations of alternative strategies to manage it in tree fruit orchards have focused on measures at the crop edges, including insecticide applications around the orchard perimeter [23,24,25] and pheromone-based attract-and-kill stations at intervals around the perimeter [25,26,27]. Among other reasons, these tactics are intended to prevent outbreaks of secondary pests that have too often followed whole-orchard applications of broad-spectrum insecticides against *H. halys*.

Research on these alternative, perimeter-based tactics for *H. halys* management has been predicated on their ability to manage its injury throughout an entire orchard block, recognizing that pest pressure may sometimes exceed their capacity to do so without additional intervention. Therefore, pheromone traps deployed at the edges and in the interior of orchards are used as part of these strategies, providing timely information on relative *H. halys* pressure that can inform grower decisions about whether and when to intervene more aggressively. For example, Short et al. [28] developed a provisional action threshold for *H. halys* that was based on cumulative captures of adults in individual pheromone-baited pyramid traps. Subsequently, Acebes-Doria et al. [21] compared *H. halys* captures in pheromone-baited pyramid traps and double-sided sticky panel traps mounted atop wooden stakes and found that while more *H. halys* were captured in pyramid traps than on sticky panels, captures between them were significantly correlated across a range of *H. halys* population densities, and both traps reflected the same temporal changes in *H. halys* seasonal phenology. Calibration of the *H. halys* action threshold for sticky traps is ongoing (T.C. Leskey).

The use of information from pheromone traps is fundamental to the success of perimeter-based *H. halys* management strategies for protecting crops throughout the planting [23,24,25]. However, despite the general belief that *H. halys* from woodland borders may pose the greatest risk to crops, the relative effects of woodlands and other habitats bordering orchards on captures in traps and, most importantly, fruit injury, have not been directly compared at the orchard block level. Here, we report studies conducted in commercial apple and peach orchards in the Mid-Altantic, USA, that compared, (1) seasonal captures of *H. halys* adults and nymphs in pheromone-baited pyramid traps deployed at orchard borders adjacent to woodland and other habitats and (2) crop injury at harvest at these borders on a block-by-block basis.

## 2. Materials and Methods 

### 2.1. Orchard Sites

Commercial apple and peach orchard blocks in Virginia (VA), West Virginia (WV), and Maryland (MD) were selected for studies in 2013 and 2014 (Table 1). In 2013 and 2014, 5 apple blocks in VA and 4 in WV/MD were used, and in both years, there were 5 peach blocks between WV and MD. Seven of the 9 apple blocks and all peach blocks were used in both years, while 1 apple block in each of VA and WV differed between years. The size (ha) of apple and peach blocks, respectively, was 4.12 ± 0.54 SE and 3.0 ± 0.71 SE. Fifteen of the orchards were bordered on 1 or 2 sides by unmanaged woodland and by 2 or 3 different habitats along the other edges (Table 1), and 1 apple block had woodland along 3 borders. Non-woodland border habitats included another orchard block, row crops (small fruit, vegetables), field crops (corn, soybean), fallow field, and buildings (homes, barns, etc.). Due to the documented importance of field crops in the movement of *H. halys* in the landscape [14,15], field crops were retained as a separate category even though there were a low number of borders classified as such. The remaining borders with fallow fields, buildings, and row crops were qualitatively different from the other types of edges, so were combined into an “other” category.

Among the apple and peach blocks, there were a variety of cultivars, and as is typical for this region, most individual blocks were comprised of 2 or more cultivars. For apples, at least 1 cultivar per block matured and was harvested in mid -September or thereafter. Similarly, each peach block contained at least 1 cultivar harvested in late July.

### 2.2. Halyomorpha halys Monitoring

Black, corrugated plastic pyramid traps (1.22 m in height, Dead-Inn, AgBio Inc., Westminster, CO, USA) were used for all studies. An inverted clear, plastic jar mounted atop each trap and ventilated by screened sections on all sides served as the collection container. Suspended from the top within each jar, a grey rubber septum containing 10.7 mg of the *H. halys* aggregation pheromone [29,30], (3*S*,6*S*,7*R*,10*S*)-10,11-epoxy-1-bisabolen-3-ol and (3*R*,6*S*,7*R*,10*S*)-10,11-epoxy-1-bisabolen-3-ol, and a commercial lure (AgBio Inc., Westminster, CO, USA) containing ~66 mg of methyl (2*E*, 4*E*, 6*Z*)-decatrienoate (MDT; an *H. halys* pheromone synergist) [31] served as the attractants. One half (2.5 × 4.7 cm) of a strip of Hercon Vaportape II (Hercon Environmental, Emigsville, PA, USA) containing dichlorvos was also suspended within each jar, and served as the killing agent. Septa and dichlorvos strips were replaced at 14-day intervals and MDT lures were replaced monthly during the sampling period.

In each orchard block, 1 trap was deployed between 2 trees at the approximate mid-point of each border row (4 traps per block), and captures of *H. halys* adults and nymphs in each trap were recorded weekly during the sampling period. In 2013, traps were deployed in apple blocks between 15 April (WV/MD) and 14 May (VA) until 17 September–10 October, according to the grower’s planned harvest date for the cultivar to be evaluated for injury. In 2014, traps in apple blocks were deployed between 7 April (WV/MD) and 22 May (VA), and monitored through 22 September–17 October at the latest. In peach blocks, traps were deployed between 17 and 25 April in 2013 and between 7 and 17 April in 2014, and monitored weekly through 28 August–12 September in 2013 and 11 August–9 October in 2014.

### 2.3. Fruit Injury Assessments

Fruit sampling for assessment of *H. halys* injury at harvest was conducted according to the harvest date scheduled by cooperating growers. In 2013, peaches were collected between 17 and 25 July, and apples between 9 September and 7 October. In 2014, peaches were sampled between 17 and 25 July, and apples between 15 September and 17 October. For peaches, samples of 10 fruit per tree were taken from the 3 trees closest to the pheromone trap at the mid-point of each border row (120 fruit per orchard) in both years. In 2013, the same protocol was used to sample apples. In 2014, to enable a comparison of fruit injury from trees near and away from the pheromone-baited traps, 10 apples per tree were also collected from an additional 4 trees in each border row, at distances of 25 and 50 m on either side of the trap in each row (280 fruit per orchard). The incidence of *H. halys* feeding injury on the surface of each fruit was determined from the presence or absence of discolored depressions on apples and deformed or discolored depressions on peaches [32]. 

### 2.4. Statistical Analysis

A generalized model framework was used to analyze *H. halys* captures and fruit injury individually for each crop type, field, and year after initial full models revealed significant effects of each. Models were based on a quasipoisson distribution with log-link function after an initial model check revealed problems with overdispersion in the datasets [33]. Each model employed habitat type along each border (field crops, “other”, orchards, or woods) as a fixed, explanatory variable and was implemented with the function glm from the base R software [34]. For the apple damage model in 2014, adjacent trap (yes or no) and its interaction with border habitat effect were included as additional fixed, explanatory variables. Upon a significant effect from each model, Tukey HSD was used for multiple comparisons, and implemented with the function glht from the multcomp package [35]. For all statistical analyses, R Software was used, and α = 0.05.

## 3. Results

### 3.1. H. Halys Captures in Apple

**Figure 1:**
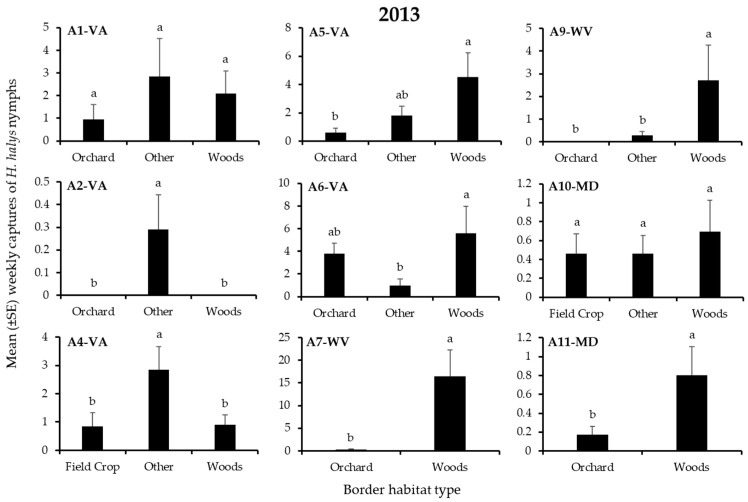
Mean (±SE) weekly captures of *H. halys* NYMPHS in pheromone-baited traps on the perimeter of 9 apple orchard blocks in the Mid-Atlantic, USA, from 15 April to 10 October 2013. Bars with shared letters are not significantly different (Tukey HSD, α = 0.05).

**Figure 2:**
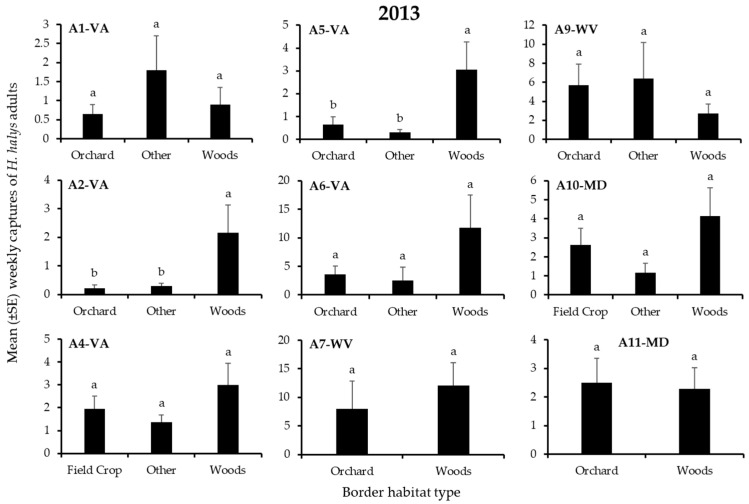
Mean (±SE) weekly captures of *H. halys* ADULTS in pheromone-baited traps on the perimeter of 9 apple orchard blocks in the Mid-Atlantic, USA, from 15 April to 10 October, 2013. Bars with shared letters are not significantly different (Tukey HSD, α = 0.05).

In 2013, 3060 *H. halys* adults and 2462 nymphs were captured in traps on the perimeter of apple blocks. Overall, 4, 25, 12, and 58% of adults, and 2, 8, 18, and 72% of nymphs were found on edges bordered by field crops, orchards, the “other” habitats, and woods, respectively. Border habitat had a significant effect on nymph captures at 7 of 9 blocks and on adult captures at 2 blocks (Table 2), but the effect of specific habitats on captures varied considerably among blocks (Figure 1 and Figure 2). Significantly highest captures of nymphs or adults occurred at only the “other” and woods borders (Figure 1 and Figure 2). Captures of nymphs were significantly highest at the “other” and woods border in 2 and 3 blocks, respectively (Figure 1), while adult captures were significantly highest at the woods border in 2 blocks (Figure 2).

**Figure 3:**
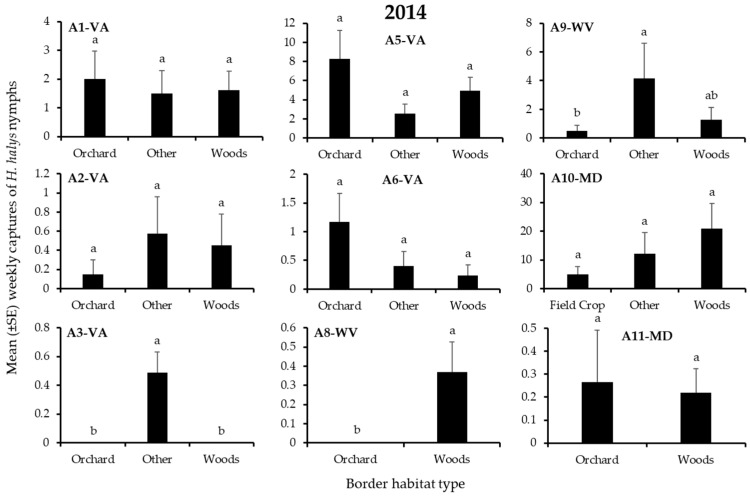
Mean (±SE) weekly captures of *H. halys* NYMPHS in pheromone-baited traps on the perimeter of 9 apple orchard blocks in the Mid-Atlantic, USA, from 7 April to 17 October 2014. Bars with shared letters are not significantly different (Tukey HSD, α = 0.05).

**Figure 4:**
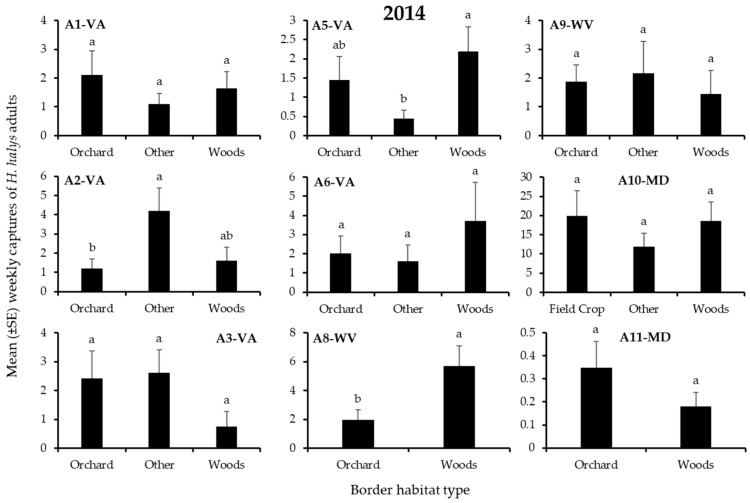
Mean (±SE) weekly captures of *H. halys* ADULTS in pheromone-baited traps on the perimeter of 9 apple orchard blocks in the Mid-Atlantic, USA, from 7 April to 17 October 2014. Bars with shared letters are not significantly different (Tukey HSD, α = 0.05).

In 2014, 3595 *H. halys* adults and 2672 nymphs were captured. For both life stages, a total of 6–17%, 10–11%, 21–26%, and 51–57% of individuals were recorded on edges bordered by field crops, orchards, “other”, or woods, respectively. Border habitat significantly affected captures of nymphs and/or adults in 3 blocks (Table 2). As in 2013, the border habitat with significantly highest nymph or adult captures varied among blocks, and again included only “other” and woods borders (Figure 3 and Figure 4). Captures of nymphs were significantly highest at the “other” or woods border in each of 1 block (Figure 3), while adult captures were significantly highest at the woods border in 1 block (Figure 4).

### 3.2. Apple Injury from H. halys

**Figure 5:**
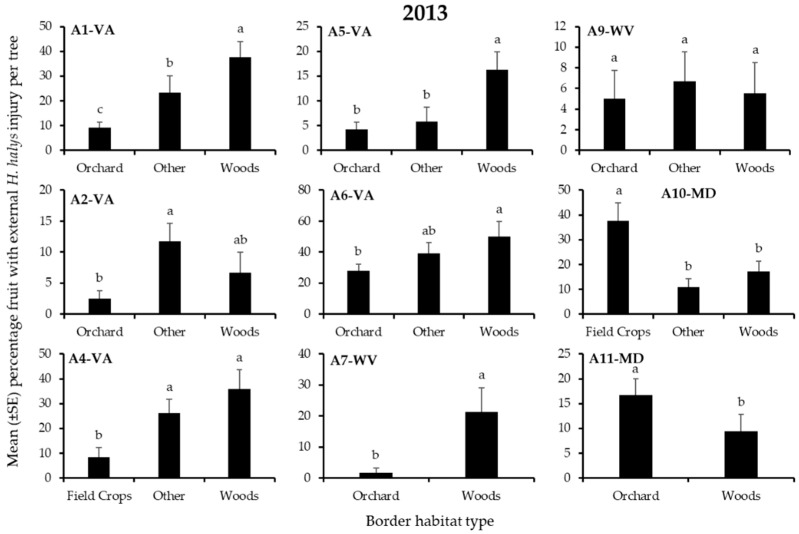
Mean (±SE) incidence of *H. halys* external injury to apples per tree at harvest (2013), from trees on the perimeter of 9 apple orchard blocks in the Mid-Atlantic, USA, that were bordered by various habitats. Because all fruit harvested in 2013 were from trees adjacent to pheromone-baited traps, injury may have been inflated. Bars with shared letters are not significantly different (Tukey HSD, α = 0.05).

In 2013, the incidence of external injury from *H. halys* to apples on the perimeter of orchard blocks was significantly affected by border habitat in 8 of the 9 blocks (Table 3). In 2 blocks, significantly highest injury was recorded from the orchard edge bordered by woods, whereas significantly highest injury occurred at the edge bordered by another orchard or field crop in each of 1 block (Figure 5). 

**Figure 6:**
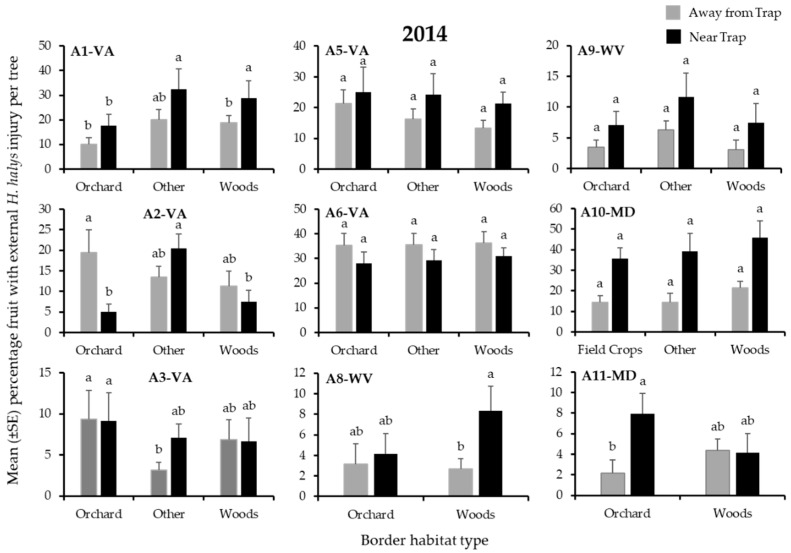
Mean (±SE) incidence of *H. halys* external injury to apples per tree at harvest (2014), from trees on the perimeter of 9 apple orchard blocks in the Mid-Atlantic, USA, that were bordered by various habitats. Black bars denote fruit samples taken from trees adjacent to pheromone-baited traps, while grey bars indicate samples taken from trees without a pheromone trap nearby. Bars with shared letters are not significantly different (Tukey HSD, α = 0.05).

By contrast, in 2014, border habitat had a significant effect on fruit injury in only 3 blocks (Table 3). The comparison of fruit injury from trees adjacent to and away from a pheromone-baited trap in 2014 showed a significant effect of proximity to the trap in 4 of 9 blocks (Table 3), and there was a significant interaction between proximity to an adjacent trap and border habitat on injury in only 2 blocks. Overall, apples sampled from trees adjacent to traps showed 2-fold more *H. halys* injury than those from trees away from traps. In 1 block (A2-VA), significantly highest fruit injury was recorded at the ”other” habitat border (Figure 6, black bars).

### 3.3. H. halys Captures in Peach

**Figure 7:**
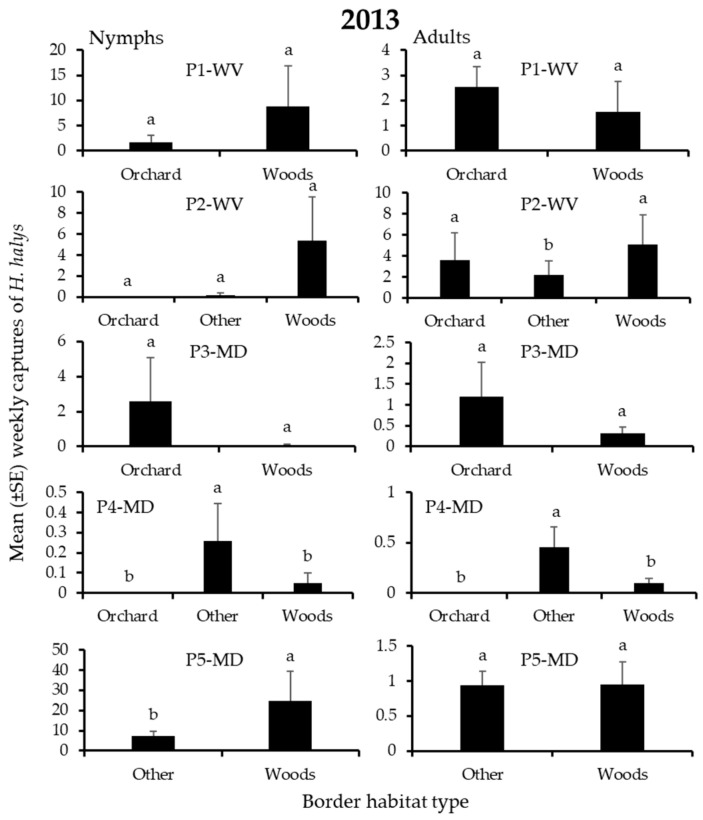
Mean (±SE) weekly trap captures of *H. halys* nymphs (left) and adults (right) in pheromone-baited traps on the perimeter of 5 peach orchard blocks in the Mid-Atlantic, USA, from 17 April to 12 September 2013. Bars with shared letters are not significantly different (Tukey HSD, α = 0.05).

In 2013, 678 *H. halys* adults, and 1605 nymphs were captured in traps on the perimeter of peach blocks. In total, 2, 15, 41, and 41% of the adults were found on edges bordered by field crops, “other”, orchards, and woods, respectively, while 0, 28, 13, and 57% of the nymphs were associated with the same habitats. For both adults and nymphs, border habitat significantly affected captures in only 1 peach block (Table 4). At 2 blocks, significantly highest nymph captures were recorded at the “other” or woods border, while adult captures were significantly highest at the “other” border in 1 block (Figure 7).

**Figure 8:**
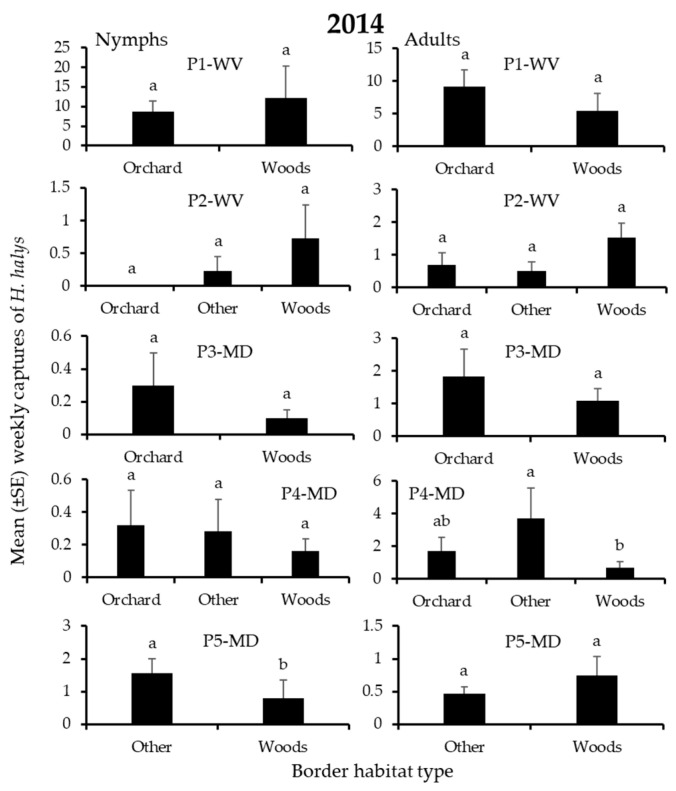
Mean (±SE) weekly trap captures of *H. halys* nymphs (left) and adults (right) in pheromone-baited traps on the perimeter of 5 peach orchard blocks in the Mid-Atlantic, USA, from 7 April to 9 October 2014. Bars with shared letters are not significantly different (Tukey HSD, α = 0.05).

In 2014, 1269 *H. halys* adults and 1156 nymphs were captured at the edges of peach blocks. In total, 3, 7, 24, and 66% of the adults were captured on edges bordered by “other”, field crops, woods, and orchards, respectively. Similarly, 1, 10, 32, and 58% of nymphs were captured at peach edges bordered by field crops, “other”, and woods, and orchards, respectively. Despite this consistent annual pattern, there was considerable block-to-block variability in captures among the border habitats (Figure 8), and border habitat significantly affected captures of adults or nymphs in each of 1 block (Table 4). Significantly highest captures of nymphs were at edges bordered by the “other” habitat in 1 block, while there were no instances of significantly highest adult captures associated with a specific border habitat (Figure 8). 

### 3.4. Peach Injury from H. halys

**Figure 9:**
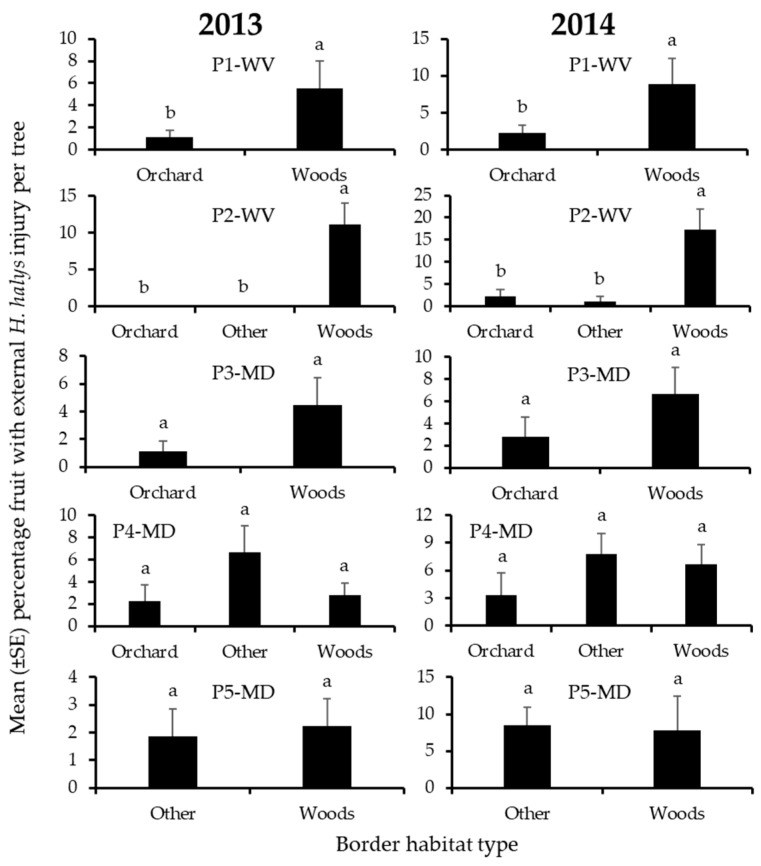
Mean (±SE) incidence of *H. halys* external injury to peaches per tree at harvest in 2013 (left) and 2014 (right), from trees on the perimeter of 5 peach orchard blocks in the Mid-Atlantic, USA, that were bordered by various habitats. Bars with shared letters are not significantly different (Tukey HSD, α = 0.05).

Injury to peaches was significantly affected by border habitat in the same 2 of the 5 blocks in 2013 and 2014 (Table 5). In both years, injury was significantly highest on edges bordered by woods in these 2 blocks (Figure 9). 

### 3.5. Border Risk Assessment Based on Highest Captures and Injury at a Common Border

In 2013, adult captures and fruit injury were significantly highest at the woods border in only 1 apple block (A5-VA), and this occurred for nymph captures and injury at the woods border in another block (A7-WV) (Table 6). By contrast, in 2014, there were no apple blocks at which significantly highest adult or nymph captures and injury occurred at the same border (Table 6).

Between 2013 and 2014, there were no instances of significantly highest adult or nymph captures and fruit injury at a common border (Table 7). 

## 4. Discussion

Substantial variability in insect captures is often inherent to pheromone-based trapping studies, particularly when monitoring spans the activity period of a bi- or multivoltine species that shows large differences in captures of each life stage at different points throughout the entire growing season, as does *H. halys* [19,21,22]. The results from this study were no exception. Several potentially important sources of variation among our orchards included pest density by orchard location, pest density by year, management practices, and biotic differences such as the plant composition of adjoining woodlands. In combination, these likely contributed to numerous instances of no statistical separations between or among border habitats in mean weekly captures on a block-by-block basis. Taking this into consideration, we nonetheless contend that overall results from these studies support the belief that woodland borders adjacent to commercial orchards in the Mid-Atlantic, USA, can, in general, be considered “riskiest” in terms of pest pressure from *H. halys*, in concurrence with the conclusion of others [7,14]. Across all orchards, 55% and 64% of adults and nymphs, respectively, were captured at orchard edges bordered by woods in 2013, and in 2014, 41% of adults and 46% of nymphs were captured where woods adjoined orchards. Moreover, between 2013 and 2014, captures of adults and/or nymphs were significantly or numerically highest at the woods border in 66.7% of apple blocks (12 of 18 blocks) and 60% of peach blocks (6 of 10 blocks), lending further support to this contention.

The influence of woodland borders on *H. halys* captures likely also pertains to other areas in the USA where *H. halys* threatens tree fruit and other vulnerable crops grown next to woodlands, especially where such woodlands are composed of similar deciduous plant hosts of *H. halys* as occur in the Mid-Atlantic region [12] and surrounding areas [7]. Moreover, visual surveys in pear orchards in northern Italy revealed highest counts of *H. halys* nymphs and adults at orchard edges bordered by “hedges” containing a number of wild hosts of *H. halys* [5], and fewest adults and nymphs in the orchard interior.

However, the present study also revealed numerous instances of highest captures of adults or nymphs at non-woodland borders, and several possible factors may account for this. First, although the effects of individual wild plant species and the plant species composition of woodland borders on *H. halys* population growth and density remain poorly understood, differences among these woodlands may have been important. For example, those containing a greater abundance or diversity of suitable *H. halys* hosts may have supported more rapid population growth than those with a less suitable host plant composition, resulting in site-specific differences in captures between woodland and non-woodland borders. Indeed, Acebes-Doria et al. [36] showed that tree hosts varied in their suitability for *H. halys* nymphal development and survivorship and that developmental rate and survivorship was significantly improved on a diet of mixed hosts. Although the specific crop in the orchard blocks adjoining the blocks in which studies were conducted was not recorded, highest captures occurred at the borders adjacent to other orchards at some sites. It is notable that this was particularly evident at the peach sites, despite typically more aggressive *H. halys* management in peaches than in lower value apples and that compared with apples [36,37], peaches are known to be a highly suitable host for *H. halys* [36].

The “other” border category, at which highest captures occurred at some sites, is more difficult to interpret, given that it contained buildings and other specialty crops such as small fruit and vegetables. Certainly, buildings are known to harbor overwintering adult *H. halys*, often in large numbers [38], and it is possible that such buildings served as a reservoir for *H. halys* that contributed to adult captures along these borders, at least during the early portion of the season. Similarly, field crops (corn, soybean) were a less common border type, but contributed substantially to captures at some sites. In general, highest *H. halys* populations in soybean and corn tend to occur at particular points in the growing season, coincident with specific plant growth stages [39], and the movement of *H. halys* from them into orchards likely occurred during only a portion of the season.

Finally, the effects of *H. halys* management and different management programs among the orchards, cannot be discounted. In general, woodlands bordering orchards would not have been treated with insecticides, although one product labelled for that purpose was available. Thus, *H. halys* population growth in woodland borders would not have been impeded as it likely was, to varying degrees, among the non-woodland borders, according to the extent of insecticide applications. Following the outbreak in the Mid-Atlantic region, there was a general increase in the number of insecticide applications to orchards, but also significant variation among growers in application frequency and the products used [2,40], based at least in part on whether fruit were destined for processing or the fresh market. This variation may also partially explain differences among sites. As mentioned previously, *H. halys* management in peaches was typically more aggressive than in apples, particularly in the years following the 2010 outbreak, when some growers in this region lost most of their peach crop [2]. While there were substantial differences among orchards in mean weekly *H. halys* captures each year, in general, adult captures in peach blocks (Figure 7 and Figure 8) were less than in apples (Figure 2 and Figure 4). These results may suggest that peach, a highly suitable host for *H. halys* [36], may be more competitive with pheromone-baited traps than apple, a less suitable host [36,37]. Given that the trapping area serviced by a single pheromone-baited trap (the area over which a single trap can reliably capture foraging insects) was reduced by >80% in an apple orchard compared with an open field where no host plants were present [41], similar studies in peach orchards to define trapping area are warranted.

At harvest in 2014, our inclusion of additional apple samples from trees away from the traps along each border was to address the question of whether the presence of a pheromone-baited trap influenced fruit injury in adjacent trees. Overall, we recorded twice as much external injury to fruit from trees adjacent to traps compared with those from trees at ≥25 m away from traps. In many, but not all instances, external injury to apples was higher in trees adjacent to traps, although most orchards showed no statistical separation between trees next to or away from traps, regardless of border habitat. Subsequently, Short et al. [28] reported that fruit sampled from trees ≥ 10 m away from pheromone-baited traps sustained less injury than fruit from trees next to traps [28], and Morrison et al. [26] revealed the behavioral basis for this by showing that the duration of adult *H. halys* retention in apple trees baited with its pheromone and pheromone synergist was 6-fold greater than in unbaited trees, resulting in more injured fruit from baited than unbaited trees. 

These results are intended to inform ongoing research toward refining and improving the effectiveness of *H. halys* monitoring and management strategies at the orchard perimeter, particularly in terms of the relative “riskiness” of different bordering habitats. With respect to interpreting the risk of fruit injury in relation to border habitat, it is informative to consider all blocks at which there was an association between numerically or significantly highest captures of adults or nymphs and numerically or significantly highest fruit injury at a common border. Between 2013 and 2014, this occurred in 14 of 18 apple blocks (7 woods, 4 “other”, 3 orchard) and in 8 of 10 peach blocks (5 woods, 3 “other”). In concurrence, Maistrello et al. [5] reported a significant correlation between visual counts of *H. halys* during the season, which were highest at borders adjacent to “hedges” with wild hosts, and injury to pears at harvest.

While our findings indicated that highest captures at a particular border were often associated with an increased likelihood of highest injury at the same border, they were based on season-long trapping. Thus, their utility to inform timely management decisions by growers is limited. Rather, the use of a prescribed action threshold based on cumulative adult captures [25,28] is a more practical application of *H. halys* monitoring results. Using inclusion cages, Acebes-Doria et al. [13] showed that while both *H. halys* nymphs and adults caused economic injury to apples and peaches, injury from adults was highest. Moreover, *H. halys* nymphs are more susceptible to insecticides than adults [42,43], suggesting that monitoring data for adults would be most appropriate to inform management decisions. The present study suggests that, in general, pheromone traps deployed at orchard edges adjacent to woodlands would be more likely to result in captures that exceed prescribed action thresholds than those deployed adjacent to other border habitats. Regardless of where traps are placed around the orchard perimeter, they would likely exceed the action threshold much more frequently during the late season (approximately mid-August onward) than earlier, due to typically highest *H. halys* populations and weekly captures in late season [19,22]. Indeed, as shown by Short et al. [28], late season captures may exceed the threshold almost every week, thereby reducing the practical value of continued monitoring during that period. Thus, monitoring at borders with wooded edges during the earlier parts of the growing season may have greatest utility for growers by providing an ongoing indication of changes in *H. halys* pressure and the need for timely intervention when the threshold is exceeded.

Importantly, while captures and injury suggested that perimeter-based practices, such as border row sprays [23,24,25] and attract and kill [25,27], may often prove to be most impactful at the orchard–woodland interfaces, non-woods border habitats also contributed frequently to substantial risk of crop injury. Given that the effects of non-woods borders on captures and fruit injury varied among border types and individual orchards, and that in many cases injury was not predicted by highest captures of either adults or nymphs, perimeter-based interventions against *H. halys* should be practiced along all borders. Yet, these findings may suggest that, in general, certain of these tactics might be modified to increase their benefits beyond those already demonstrated [25,27]. For example, deploying more attract-and-kill stations along the “riskiest” border(s) might be expected to enhance the mitigation of risk from these borders. In general, an increased density of attract-and-kill stations along woodland borders seems most warranted, although individual grower experience with crop injury along different orchard borders could also inform the use of this tactic. Fruit injury on apple trees adjacent to a pheromone trap was often higher than on trees away from traps, conforming to the findings of other studies [26,28]. To mitigate this effect, pheromone-based attract-and-kill stations may be best deployed at some distance from the orchard edge, for example between the orchard and the adjoining border habitat. The multiple benefits of an attract-and-kill tactic for *H, halys* using pheromone lures in association with insecticide-impregnated netting has been discussed [25] and is under evaluation. 

## 5. Conclusions

Seasonal captures of *H. halys* and its injury to apples and peaches in the Mid-Atlantic, USA, were often highest where orchard edges adjoined unmanaged woodlands, although other habitats bordering orchards, including field crops, the “other” habitats, and other orchard blocks also contributed substantially to captures and fruit injury at harvest in some instances. Cumulative captures of adult *H. halys* in pheromone-baited monitoring traps to inform management decisions would likely exceed prescribed action thresholds more frequently when traps are deployed at the orchard–woodland interface. Similarly, the impact *of H. halys* management tactics focused on the orchard perimeter, including perimeter sprays or attract and kill, may be optimized by concentrating on orchard edges where risk is highest. In many, but not all instances, highest risk was associated with woodland borders. Pheromone traps deployed in orchard perimeter rows often resulted in higher injury to fruit in trees adjacent to traps compared with trees away from traps, suggesting that this effect might be mitigated by some degree of physical separation between pheromone-based attract-and-kill stations and the orchard edge.

## Figures and Tables

**Table 1:** Physical characteristics of commercial apple and peach orchard blocks in Virginia, West Virginia, and Maryland used to assess the effects of border habitat on *H. halys* captures and fruit injury at harvest.

Crop	State	Year(s)	Block Code	Area (ha)	Frequency of Border Habitats			
					Woods	Another Orchard	Field Crop	Other Habitats ^1^
apple	Virginia	2013–2014	A1-VA	7.31	2	1	0	1
apple	Virginia	2013–2014	A2-VA	3.28	1	1	0	2
apple	Virginia	2014	A3-VA	4.27	1	2	0	1
apple	Virginia	2013	A4-VA	5.27	2	1	1	0
apple	Virginia	2013–2014	A5-VA	4.70	2	1	0	1
apple	Virginia	2013–2014	A6-VA	4.43	1	2	0	1
apple	West Virginia	2013	A7-WV	6.0	3	1	0	0
apple	West Virginia	2014	A8-WV	3.7	2	1	0	0
apple	West Virginia	2013–2014	A9-WV	4.4	1	2	0	1
apple	Maryland	2013–2014	A10-MD	1.6	2	0	1	1
apple	Maryland	2013–2014	A11-MD	1.0	1	2	0	1
peach	West Virginia	2013–2014	P1-WV	4.0	1	3	0	0
peach	West Virginia	2013–2014	P2-WV	5.2	2	1	0	1
peach	Maryland	2013–2014	P3-MD	1.5	2	1	0	1
peach	Maryland	2013–2014	P4-MD	2.2	2	1	0	1
peach	Maryland	2013–2014	P5-MD	1.3	1	1	0	2

^1^ Other includes small fruit, vegetables, human-made structures, and fallow field.

**Table 2:** Summary of statistical results for captures of *H. halys* nymphs and adults in pheromone-baited traps on the edges of 9 apple blocks per year (2013 and 2014) in the Mid-Atlantic, USA. Significant *p* values are highlighted in bold font.

Block	Factor	2013			2014		
		df	*χ^2^*	*p*	df	*χ^2^*	*p*
*Nymphs*							
A1-VA	Border	2	1.22	0.54	2	0.17	0.92
A2-VA	Border	2	9.53	**0.01**	2	0.93	0.63
A3-VA	Border	-	-	-	2	30.3	**0.0001**
A4-VA	Border	2	6.30	**0.04**	-	-	-
A5-VA	Border	2	6.49	**0.04**	2	4.27	0.12
A6-VA	Border	2	6.78	**0.03**	2	3.54	0.17
A7-WV	Border	1	5.76	**0.02**	1	-	-
A8-WV	Border	1	-	-	1	4.22	**0.04**
A9-WV	Border	2	26.9	**0.0001**	2	6.01	**0.05**
A10-MD	Border	2	0.45	0.80	2	2.67	0.26
A11-MD	Border	1	5.67	**0.02**	1	0.04	0.85
*Adults*							
A1-VA	Border	2	1.80	0.41	2	1.00	0.61
A2-VA	Border	2	19.7	**0.0001**	2	6.34	**0.04**
A3-VA	Border	-	-	-	2	3.45	0.18
A4-VA	Border	2	4.63	0.10	-	-	-
A5-VA	Border	2	7.99	**0.02**	2	5.74	**0.05**
A6-VA	Border	2	4.91	0.09	2	1.27	0.53
A7-WV	Border	1	0.34	0.56	-	-	-
A8-WV	Border	1	-	-	1	3.12	**0.05**
A9-WV	Border	2	1.20	0.55	2	0.34	0.84
A10-MD	Border	2	3.47	0.18	2	1.18	0.55
A11-MD	Border	1	0.04	0.85	1	1.82	0.18

**Table 3:** Summary of statistical results for the incidence of external fruit injury from *H. halys* in 9 apple blocks per year (2013 and 2014) in the Mid-Atlantic, USA. Significant *p* values are highlighted in bold font.

Block	Factor	df	*χ^2^*	*p*
*2013*				
A1-VA	Border	2	38.6	**0.0001**
A2-VA	Border	2	21.2	**0.0001**
A4-VA	Border	2	30.1	**0.0001**
A5-VA	Border	2	17.1	**0.001**
A6-VA	Border	2	16.9	**0.001**
A7-WV	Border	1	18.2	**0.0001**
A9-WV	Border	2	0.31	0.86
A10-MD	Border	2	27.1	**0.0001**
A11-MD	Border	1	4.17	**0.04**
*2014*				
A1-VA	Border	2	8.54	**0.01**
A1-VA	Adjacent Trap	1	3.63	0.06
A1-VA	Interaction	2	0.17	0.92
A2-VA	Border	2	4.49	0.11
A2-VA	Adjacent Trap	1	13.9	**0.0002**
A2-VA	Interaction	2	19.5	**0.0001**
A3-VA	Border	2	8.49	**0.01**
A3-VA	Adjacent Trap	1	0.00	0.95
A3-VA	Interaction	2	3.11	0.21
A5-VA	Border	2	4.78	0.09
A5-VA	Adjacent Trap	1	0.51	0.47
A5-VA	Interaction	2	1.06	0.59
A6-VA	Border	2	0.04	0.98
A6-VA	Adjacent Trap	1	3.50	0.06
A6-VA	Interaction	2	0.12	0.94
A8-WV	Border	2	0.73	0.39
A8-WV	Adjacent Trap	1	**10.2**	**0.001**
A8-WV	Interaction	2	1.39	0.24
A9-WV	Border	2	2.45	0.29
A9-WV	Adjacent Trap	1	3.80	0.05
A9-WV	Interaction	2	0.14	0.93
A10-MD	Border	2	6.41	**0.04**
A10-MD	Adjacent Trap	1	18.4	**0.0001**
A10-MD	Interaction	2	1.12	0.57
A11-MD	Border	2	3.46	0.06
A11-MD	Adjacent Trap	1	8.86	**0.003**
A11-MD	Interaction	2	4.97	**0.03**

**Table 4:** Summary of statistical results for captures of *H. halys* nymphs and adults in pheromone-baited traps on the edges of 5 peach blocks in the Mid-Atlantic, USA, in 2013 and 2014. Significant *p* values are highlighted in bold font.

Block	Factor	2013			2014		
		df	*χ^2^*	*p*	df	*χ^2^*	*p*
*Nymphs*							
P1-WV	Border	1	2.14	0.14	1	0.30	0.58
P2-WV	Border	2	3.98	0.08	2	3.17	0.20
P3-MD	Border	1	2.33	0.13	1	1.33	0.25
P4-MD	Border	2	3.03	0.22	2	0.83	0.66
P5-MD	Border	1	4.31	**0.04**	1	4.31	**0.04**
*Adults*							
P1-WV	Border	1	0.45	0.50	1	0.76	0.38
P2-WV	Border	2	0.69	0.71	2	4.27	0.12
P3-MD	Border	1	1.68	0.19	1	0.72	0.39
P4-MD	Border	2	8.40	**0.02**	2	6.01	**0.05**
P5-MD	Border	1	0.00	0.97	1	0.002	0.97

**Table 5:** Summary of statistical results for incidence of external fruit injury from *H. halys* on the perimeter of 5 peach blocks in 2013 and 2014 in the Mid-Atlantic USA. Significant *p* values are highlighted in bold font.

Block	Factor	2013			2014		
		df	*χ^2^*	*p*	df	*χ^2^*	*p*
P1-WV	Border	1	5.38	**0.02**	1	4.52	**0.03**
P2-WV	Border	2	39.4	**0.0001**	2	16.9	**0.0002**
P3-MD	Border	1	2.98	0.08	1	1.64	0.20
P4-MD	Border	2	3.54	0.17	2	1.61	0.45
P5-MD	Border	1	0.02	0.88	1	0.02	0.88

**Table 6:** Qualitative comparisons of *H. halys* adult and nymph captures and fruit injury at harvest by orchard border habitat in apple blocks in the Mid-Atlantic, USA, in 2013 and 2014.

Block	2013			2014		
	Adults ^1^	Nymphs	Injury	Adults	Nymphs	Injury
A1-VA	Other	Other	**Woods**	Orchard	Orchard	Other
A2-VA	**Woods**	**Other**	Other	Other	Other	**Other**
A3-VA	-	-	-	Other	Other	Orchard
A4-VA	Woods	Other	Woods	-	-	-
A5-VA	Woods *	Woods	Woods *	Woods	Orchard	Orchard
A6-VA	Woods	Woods	Woods	Woods	Orchard	Woods
A7-WV	Woods	Woods *	Woods *	-	-	-
A8-WV	-	-	-	**Woods**	**Woods**	Woods
A9-WV	Other	Other	Other	Other	Other	Other
A10-MD	Woods	Woods	**Field Crop**	Field Crop	Woods	Woods
A11-MD	Orchard	**Woods**	**Orchard**	Orchard	Orchard	Orchard

^1^ Bolded adults, nymphs, or injury indicate captures or injury, respectively, that were significantly highest at the specified border in each orchard block (GLM, α = 0.05). Non-bolded adults, nymphs, or injury indicate captures or injury, respectively, that were numerically highest at the specified border in each orchard block. * denotes a match between significantly highest captures and significantly highest injury at the same border.

**Table 7:** Qualitative comparisons of *H. halys* adult and nymph captures and fruit injury at harvest by orchard border habitat in peach blocks in the Mid-Atlantic, USA, in 2013 and 2014.

Block	2013			2014		
	Adults ^1^	Nymphs	Injury	Adults	Nymphs	Injury
P1-WV	Orchard	Woods	**Woods**	Orchard	Woods	**Woods**
P2-WV	Woods	Woods	**Woods**	Woods	Woods	**Woods**
P3-MD	Orchard	Orchard	Woods	Orchard	Orchard	Woods
P4-MD	**Other**	**Other**	Other	Other	Orchard	Other
P5-MD	Woods	**Woods**	Woods	Woods	**Other**	Other

^1^ Bolded adults, nymphs, or injury indicate captures or injury, respectively, that were significantly highest at the specified border in each orchard block (GLM, α = 0.05). Non-bolded adults, nymphs, or injury indicate captures or injury, respectively, that were numerically highest at the specified border in each orchard block.

## Data Availability

There are no publicly accessible archives of the data.

## References

[1] Leskey T.C., Nielsen A.L. (2018). Impact of the invasive brown marmorated stink bug in North America and Europe: History, biology, ecology, and management. Annu. Rev. Entomol..

[2] Leskey T.C., Short B.D., Butler B.R., Wright S.E. (2012). Impact of the invasive brown marmorated stink bug, *Halyomorpha halys* (Stål), in mid-Atlantic tree fruit orchards in the United States: Case studies of commercial management. Psyche.

[3] Leskey T.C., Lee D.-H., Short B.D., Wright S.E. (2012). Impact of insecticides on the invasive *Halyomorphahalys* (Hemiptera: Pentatomidae): Analysis of insecticide lethality. J. Econ. Entomol..

[4] Bosco L., Moraglio S.T., Tavella L. (2017). *Halyomorpha halys*, a serious threat for hazelnut in newly invaded areas. J. Pest. Sci..

[5] Maistrello L., Vaccari G., Caruso S., Costi E., Bortolini S., Macavei L., Foca G., Ulrici A., Bortolotti P.P., Nannini R. (2017). Monitoring of the invasive *Halyomorpha halys*, a new key pest of fruit orchards in northern Italy. J. Pest. Sci..

[6] Nielsen A.L., Hamilton G.C. (2009). Life history of the invasive species *Halyomorpha halys* (Hemiptera: Pentatomidae) in northeastern United States. Ann. Entomol. Soc. Am..

[7] Bakken A.J., Schoof S.C., Bickerton M., Kamminga K.L., Jenrette J.C., Malone S., Abney M.A., Herbert D.A., Reisig D., Kuhar T.P. (2015). Occurrence of brown marmorated stink bug (Hemiptera: Pentatomidae) on wild hosts in nonmanaged woodlands and soybean fields in North Carolina and Virginia. Environ. Entomol..

[8] Bergmann E.J., Venugopal P.D., Martinson H.M., Raupp M.J., Shrewsbury P.M. (2016). Host plant use by the invasive *Halyomorpha halys* (Stål) on woody ornamental trees and shrubs. PLoS ONE.

[9] Lee D.-H., Nielsen A.L., Leskey T.C. (2014). Dispersal capacity and behavior of nymphal stages of *Halyomorpha halys* (Hemiptera: Pentatomidae) evaluated under laboratory and field conditions. J. Insect. Behav..

[10] Wiman N.G., Walton V.M., Shearer P.W., Rondon S.I., Lee J.C. (2014). Factors affecting flight capacity of brown marmorated stink bug, *Halyomorpha halys* (Hemiptera: Pentatomidae). J. Pest. Sci..

[11] Lee D.-H., Leskey T.C. (2015). Flight behavior of foraging and overwintering brown marmorated stink bug, *Halyomorpha halys* (Hemiptera: Pentatomidae). Bull. Entomol. Res..

[12] Acebes-Doria A.L., Leskey T.C., Bergh J.C. (2017). Temporal and directional patterns of nymphal *Halyomorpha halys* (Hemiptera: Pentatomidae) movement on the trunk of selected wild and tree fruit hosts in the Mid-Atlantic region. Environ. Entomol..

[13] Acebes-Doria A.L., Leskey T.C., Bergh J.C. (2016). Injury to apples and peaches at harvest from feeding by *Halyomorpha halys* (Stål) (Hemiptera: Pentatomidae) nymphs early and late in the season. Crop Prot..

[14] Venugopal P.D., Coffey P.L., Dively G.P., Lamp W.O. (2014). Adjacent habitat influence on stink bug (Hemiptera: Pentatomidae) densities and associated damage at field corn and soybean edges. PLoS ONE.

[15] Venugopal P.D., Martinson H.M., Bergmann E.J., Shrewsbury P.M., Raupp M.J. (2015). Edge effects influence the abundance of the invasive *Halyomorpha halys* (Hemiptera: Pentatomidae) in woody plant nurseries. Environ. Entomol..

[16] Joseph S.V., Stallings J.W., Leskey T.C., Krawczyk G., Polk D., Butler B., Bergh J.C. (2014). Spatial distribution of brown marmorated stink bug injury at harvest mid-Atlantic in apple orchards. J. Econ. Entomol..

[17] Joseph S.V., Bergh J.C., Wright S.E., Leskey T.C. (2013). Factors affecting captures of brown marmorated stink bug, *Halyomorpha halys* (Hemiptera: Pentatomidae), in baited pyramid traps. J. Entomol. Sci..

[18] Morrison W.R., Cullum J.P., Leskey T.C. (2015). Evaluation of trap designs and deployment strategies for capturing *Halyomorpha halys* (Hemiptera: Pentatomidae). J. Econ. Entomol..

[19] Leskey T.C., Agnello A., Bergh J.C., Dively G.P., Hamilton G.C., Jentsch P., Khrimian A., Krawczyk G., Kuhar T.P., Lee D.-H. (2015). Attraction of the invasive Halyomorpha halys (Hemiptera: Pentatomidae) to traps baited with semiochemical stimuli across the United States. Environ. Entomol..

[20] Rice K.B., Morrison W.R., Short B.D., Acebes-Doria A.L., Bergh J.C., Leskey T.C. (2018). Improved trap designs and retention mechanisms for *Halyomorpha halys* (Hemiptera: Pentatomidae). J. Econ. Entomol..

[21] Acebes-Doria A.L., Morrison W.R., Short B.D., Rice K.B., Bush H.G., Kuhar T.P., Duthie C., Leskey T.C. (2018). Monitoring and biosurveillance tools for the brown marmorated stink bug, *Halyomorpha halys* (Stål) (Hemiptera: Pentatomidae). Insects.

[22] Acebes-Doria A.L., Agnello A.M., Alston D.G., Andrews H., Beers E.H., Bergh J.C., Bessin R., Blaauw B.R., Buntin G.D., Burkness E.C. (2020). Season-long monitoring of the brown marmorated stink bug (Hemiptera: Pentatomidae) throughout the United States using commercially available traps and lures. J. Econ. Entomol..

[23] Blaauw B.R., Polk D., Nielsen A.L. (2015). IPM-CPR for peaches: Incorporating behaviorally-based methods to manage *Halyomorpha halys* and key pests in peach. Pest Manag. Sci..

[24] Akotsen-Mensah C., Blaauw B., Short B., Leskey T.C., Bergh J.C., Polk D., Nielsen A.L. (2020). Using IPM-CPR as a management program for apple orchards. J. Econ. Entomol..

[25] Leskey T.C., Short B.D., Ludwick D. (2020). Comparison and refinement of integrated pest management tactics for *Halyomorpha halys* (Hemiptera: Pentatomidae) management in apple orchards. J. Econ. Entomol..

[26] Morrison W.R., Lee D.-H., Short B.D., Khrimian A., Leskey T.C. (2016). Establishing the behavioral basis for an attract-and-kill strategy to manage the invasive *Halyomorpha halys* in apple orchards. J. Pest Sci..

[27] Morrison W.R., Blaauw B.R., Short B.D., Nielsen A.L., Bergh J.C., Krawczyk G., Park Y.-L., Butler B., Khrimian A., Leskey T.C. (2018). Successful management of *Halyomorpha halys* (Hemiptera: Pentatomidae) in commercial apple orchards with an attract-and-kill strategy. Pest Manag. Sci..

[28] Short B.D., Khrimian A., Leskey T.C. (2016). Pheromone-based decision support tools for management of *Halyomorpha halys* in apple orchards: Development of a treatment threshold. J. Pest Sci..

[29] Leskey T.C., Khrimian A., Weber D.C., Aldrich J.C., Short B.D., Lee D.-H., Morrison W.R. (2015). Behavioral responses of the invasive Halyomorpha halys (Stål) to traps baited with stereoisomeric mixtures of 10,11-Epoxy-1-bisabolen-3-OL. J. Chem. Ecol..

[30] Khrimian A., Zhang A., Weber D.C., Ho H.Y., Aldrich J.R., Vermillion K.E., Siegler M.A., Shirali S., Guzman F., Leskey T.C. (2014). Discovery of the aggregation pheromone of the brown marmorated stink bug (Halyomorpha halys) through the creation of stereoisomeric libraries of 1-bisabolen-3-ols. J. Nat. Prod..

[31] Weber D.C., Leskey T.C., Walsh G.C., Khrimian A. (2014). Synergy of aggregation pheromone with methyl (*E,E,Z*)-2,4,6-decatrienoate in attraction of *Halyomorpha halys* (Hemiptera: Pentatomidae). J. Econ. Entomol..

[32] Joseph S.V., Nita M., Leskey T.C., Bergh J.C. (2015). Temporal effects on the incidence and severity of brown marmorated stink bug (Hemiptera: Pentatomidae) feeding injury to peaches and apples during the fruiting period in Virginia. J. Econ. Entomol..

[33] Aho K.A. (2014). Foundational and Applied Statistics for Biologists Using R.

[34] R Core Team (2020). R: A Language and Environment for Statistical Computing.

[35] Hothorn T., Bretz F., Westfall P., Heiderger R.M., Schuetzenmeister A., Scheibe S. (2020). Package ‘Multcomp’. https://cran.r-project.org/web/packages/multcomp/multcomp.pdf.

[36] Acebes-Doria A.L., Leskey T.C., Bergh J.C. (2016). Host plant effects on *Halyomorpha halys* (Hemiptera: Pentatomidae) nymphal development and survivorship. Environ. Entomol..

[37] Funayama K. (2002). Comparison of the susceptibility to injury of apple cultivars by stink bugs. Jpn. J. Appl. Entomol. Zool..

[38] Inkley D.B. (2012). Characteristics of home invasion by the brown marmorated stink bug (Hemiptera: Pentatomidae). J. Entomol. Sci..

[39] Venugopal P.D., Dively G.P., Lamp W.O. (2015). Spatiotemporal dynamics of the invasive *Halyomorpha halys* (Hemiptera: Pentatomidae) in and between adjacent corn and soybean fields. J. Econ. Entomol..

[40] Ludwick D., Morrison W.R., Acebes-Doria A.L., Agnello A.M., Bergh J.C., Buffington M.L., Hamilton G.C., Harper J.K., Hoelmer K.A., Krawczyk G. (2020). Invasion of the brown marmorated stink bug (Hemiptera: Pentatomidae) into the USA: Developing a national response to an invasive species crisis through collaborative research and outreach efforts. J. IPM.

[41] Kilpatrick D.M., Acebes-Doria A.L., Rice K.B., Short B.D., Adams C.G., Gut L.J., Leskey T.C. (2019). Estimating monitoring trap plume reach and trapping area for nymphal and adult *Halyomorpha halys* (Hemiptera: Pentatomidae) in crop and non-crop habitats. Environ. Entomol..

[42] Nielsen A.L., Shearer P.W., Hamilton G.C. (2008). Toxicity of insecticides to *Halyomorpha halys* (Hemiptera: Pentatomidae) using glass-vial bioassays. J. Econ. Entomol..

[43] Bergmann E.J., Raupp M.J. (2014). Efficacy of common ready to use insecticides against *Halyomorpha halys* (Hemiptera: Pentatomidae). Fla. Entomol..

